# The human melanoma proteome atlas—Defining the molecular pathology

**DOI:** 10.1002/ctm2.473

**Published:** 2021-07-04

**Authors:** Lazaro Hiram Betancourt, Jeovanis Gil, Yonghyo Kim, Viktória Doma, Uğur Çakır, Aniel Sanchez, Jimmy Rodriguez Murillo, Magdalena Kuras, Indira Pla Parada, Yutaka Sugihara, Roger Appelqvist, Elisabet Wieslander, Charlotte Welinder, Erika Velasquez, Natália Pinto de Almeida, Nicole Woldmar, Matilda Marko‐Varga, Krzysztof Pawłowski, Jonatan Eriksson, Beáta Szeitz, Bo Baldetorp, Christian Ingvar, Håkan Olsson, Lotta Lundgren, Henrik Lindberg, Henriett Oskolas, Boram Lee, Ethan Berge, Marie Sjögren, Carina Eriksson, Dasol Kim, Ho Jeong Kwon, Beatrice Knudsen, Melinda Rezeli, Runyu Hong, Peter Horvatovich, Tasso Miliotis, Toshihide Nishimura, Harubumi Kato, Erik Steinfelder, Madalina Oppermann, Ken Miller, Francesco Florindi, Qimin Zhou, Gilberto B. Domont, Luiciana Pizzatti, Fábio C. S. Nogueira, Peter Horvath, Leticia Szadai, József Tímár, Sarolta Kárpáti, A. Marcell Szász, Johan Malm, David Fenyö, Henrik Ekedahl, István Balázs Németh, György Marko‐Varga

**Affiliations:** ^1^ Division of Oncology Department of Clinical Sciences Lund Lund University Lund Sweden; ^2^ Department of Pathology Semmelweis University Hungary; ^3^ Department of Dermatology Venerology and Dermatooncology Semmelweis University Budapest Hungary; ^4^ Section for Clinical Chemistry Department of Translational Medicine Lund University Skåne University Hospital Malmö Malmö Sweden; ^5^ Department of Biochemistry and Biophysics Karolinska Institute Stockholm Sweden; ^6^ Clinical Protein Science & Imaging Biomedical Centre Department of Biomedical Engineering Lerund University Lund Sweden; ^7^ Chemistry Institute Federal University of Rio de Janeiro Rio de Janeiro Brazil; ^8^ Department of Molecular Biology University of Texas Southwestern Medical Center Texas USA; ^9^ Department of Biochemistry and Microbiology Warsaw University of Life Sciences Warszawa Poland; ^10^ Department of Internal Medicine and Oncology Semmelweis University Budapest Hungary; ^11^ SUS University Hospital Lund Lund Sweden; ^12^ Department of Surgery Clinical Sciences Lund University SUS Lund Sweden; ^13^ Chemical Genomics Global Research Lab Department of Biotechnology College of Life Science and Biotechnology Yonsei University Seoul Republic of Korea; ^14^ Department of Pathology University of Utah Salt Lake City USA; ^15^ Department of Biochemistry and Molecular Pharmacology Institute for Systems Genetics New York University Grossman School of Medicine New York USA; ^16^ Department of Analytical Biochemistry Faculty of Science and Engineering University of Groningen Groningen The Netherlands; ^17^ Translational Science and Experimental Medicine Cardiovascular Renal and Metabolism IMED Biotech Unit AstraZeneca Gothenburg Sweden; ^18^ Department of Oncology St. Marianna University School of Medicine Kanagawa Japan; ^19^ Department of Surgery Tokyo Medical University Tokyo Japan; ^20^ ThermoFisher Scientific HQ San Jose California USA; ^21^ BBMRI‐ERIC HQ Graz Austria; ^22^ Department of Plastic and Reconstructive Surgery Shanghai Ninth People's Hospital Shanghai Jiao Tong University School of Medicine Shanghai P. R. China; ^23^ Synthetic and Systems Biology Unit Biological Research Center Szeged Hungary; ^24^ Department of Dermatology and Allergology University of Szeged Szeged Hungary; ^25^ Department of Bioinformatics Semmelweis University Budapest Hungary; ^26^ Department of Internal Medicine and Oncology Semmelweis University Budapest Hungary

**Keywords:** heterogeneity, histopathology, metastatic malignant melanoma, proteogenomics, subcellular localization

## Abstract

The MM500 study is an initiative to map the protein levels in malignant melanoma tumor samples, focused on in‐depth histopathology coupled to proteome characterization. The protein levels and localization were determined for a broad spectrum of diverse, surgically isolated melanoma tumors originating from multiple body locations. More than 15,500 proteoforms were identified by mass spectrometry, from which chromosomal and subcellular localization was annotated within both primary and metastatic melanoma. The data generated by global proteomic experiments covered 72% of the proteins identified in the recently reported high stringency blueprint of the human proteome. This study contributes to the NIH Cancer Moonshot initiative combining detailed histopathological presentation with the molecular characterization for 505 melanoma tumor samples, localized in 26 organs from 232 patients.

## Graphical abstract

The MM500 study is an initiative to map the protein levels in malignant melanoma tumor samples, focused on in‐depth histopathology coupled to proteome characterization. The protein levels and localization were determined for a broad spectrum of diverse melanoma tumors originating from multiple body locations. More than 15,500 proteoforms were identified by mass spectrometry, from which chromosomal and sub‐cellular localization was annotated within both primary and metastatic melanoma.

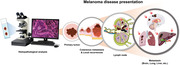

## 1 INTRODUCTION

Metastatic malignant melanoma carries a poor prognosis, however, surgical intervention of the primary melanoma is curative in most patients, which underlines the importance of early diagnosis. In 2020, Globocan reported 324,635 new cases and 57,043 deaths (https://gco.iarc.fr)^1^ from melanoma worldwide. In many European countries, melanoma is increasing at a rate of 3‐7% and this figure is expected to rise further.^2^, ^3^, ^4^ Approximately 120,000 new cases in the United States were expected to be diagnosed in 2020, with about 7000 patients dying from the disease. In Sweden, melanoma now ranks number 5‐6 among cancers (incidence: 38/100,000 in 2019) and the 10‐year survival approaches 90% as the proportion of thinner melanomas with an extremely good prognosis, increases.^5^, ^6^ Metastatic melanoma (MM) used to have an extremely poor prognosis until 10 years ago, since then the development of modern drugs modulating the immune response or targeting specific cellular signaling events has prolonged survival from months to years for many patients.^7^, ^8^, ^9^ However, some patients with MM do not fully respond or develop drug resistance to the novel treatments why there is still need for more mechanistic disease knowledge.^10^, ^11^, ^12^ Clonal diversity and genetic inter‐ and intratumor heterogeneity in MM are in particular, poorly understood.^13^, ^14^, ^15^, ^16^

Currently, there is only one clinically validated predictive marker (BRAFV600E) for molecular targeted therapy.^17^, ^18^, ^19^, ^20^ Clinically useful markers are lacking for deciding on sequential administration of drugs in first‐line management (targeted vs. immune checkpoint therapy).^21^, ^22^, ^23^ Cutting‐edge technologies (such as proteomics, single‐cell transcriptomics, spatial imaging, machine learning, etc.) have been developed to identify novel biomarkers and will contribute to building clinical prognostic and predictive useful information.^24^, ^25^, ^26^, ^27^ For example genomic research has led to major improvements in the diagnosis and treatment of cancer and the foundation of precision oncology. Nevertheless, the challenge today is still an unmet need of information guiding the daily treatment of patients. The proteome, which is defined as the entire set of proteins of a cell, tissue, organ, or body fluid in time, space as well as in a stimuli‐dependent stage is composed of proteoforms that are expressed in response to specific intrinsic and extrinsic signals. Moreover, proteins are the true functional molecular species in the cell, important both for disease modulation and drug interactions. Proteomics is defined as the characterization of proteins encoded by the genome of a given organism at a given time in a given state.^28^ By mass spectrometry (MS) a sensitive analysis of complex mixtures of proteins and peptides is enabled. MS‐based techniques provide a means of identifying and quantifying proteins, studying post‐translational modifications, protein interactions, and localization. Thus, identification of protein biomarkers could be used to evaluate the clinical course of the disease and identify protein signatures related to the effects of treatment.^29^, ^30^, ^31^ For example, some recent studies revealed that heterogeneous expression of the BRAF V600E mutated protein in melanoma patients was related to survival.^15^, ^32^

**HIGHLIGHT**

- Protein expression combined with in‐depth histopathological characterization is determined for a broad spectrum of primary and metastatic melanoma tumors isolated from multiple body locations.
- Mass spectrometry analysis provides identification of more than 15,500 proteoforms subsequently annotated to chromosomal and subcellular localization.
- The presented melanoma tumor protein blueprint cover 72% of proteins currently identified in the human proteome.

In the present study, the proteomic map of primary and MM was determined and combined with in‐depth pathophysiological and clinical characterization of the tumors. The MM500 study (505 samples from 294 tumors obtained from 232 patients) utilizes samples from both Sweden (BioMel biobank, SUS University Hospital Lund) and Hungary (Semmel study, Semmelweis University Hospital and Melszeg study, Szeged University Hospital). The overall aim of the MM500 study is to identify and define the overall melanoma proteome including the intracellular localization of expressed proteins. By combining this information with genome and transcriptome sequencing, digital pathology (DP), and clinical information, this study will serve as a roadmap to find new markers for prognostic and predictive use in malignant melanoma. The milestone delivery of the melanoma proteome will enable open resource bioinformatics tools to be applied in an interactive way with computational science.

## 2 RESULTS AND DISCUSSION

The present publication belongs to a series of two on the Human Melanoma Proteome published by Clinical and Translational Medicine. Both are integral parts of the MM500 study. The other publication is entitled “The Human Melanoma Proteome Atlas—Complementing the Melanoma Transcriptome.” It provides a map with the abundance of identified proteins, a comparison with the TCGA melanoma transcriptome data, analysis of post‐translational modifications, along with the detection of missing proteins, melanoma driver mutations at the protein level, and the analysis of protein expression in the blood plasma of melanoma patients.

### 2.1 Clinicopathological data of the MM500 cohort

Histopathological characterization of the tumor samples was made, followed by a quality assessment by detailed pathological analysis of the fresh frozen and formalin‐fixed and paraffin‐embedded (FFPE) tissue samples.

**FIGURE 1:**
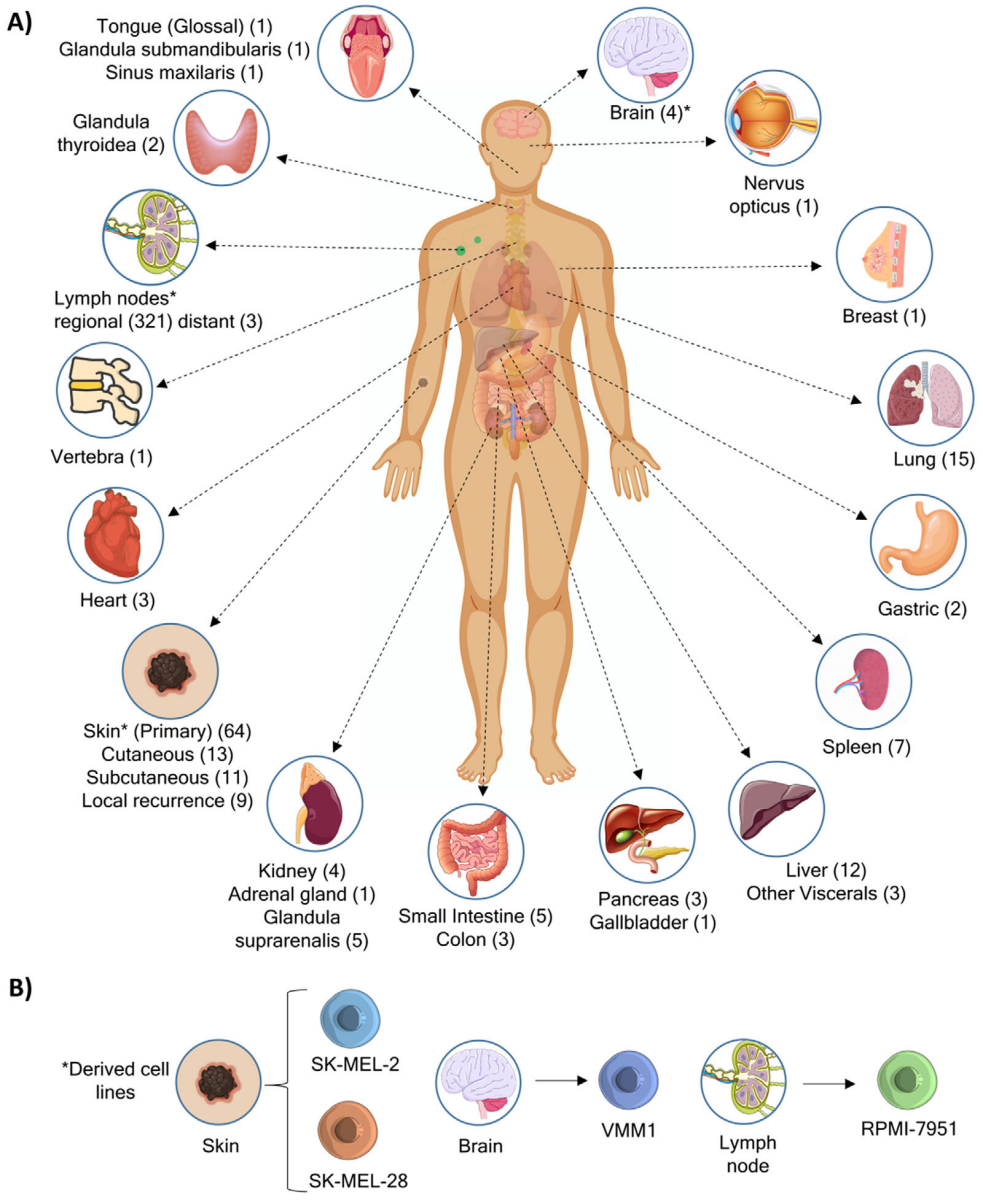
Illustration depicting (A) the sites of surgically isolated tumors included in the MM500 study and (B) specific melanoma reference cell lines. The number of tumor samples analyzed is given in parenthesis. The asterisk (*) indicates the original relationship between the tumor and derived cell lines

The body sites of the metastatic tumor specimens are outlined in Figure 1A. The cohort included 294 melanoma tumors and from most of them multiple pieces were collected and analyzed which made a total number of 505 tumor samples. The melanomas were stratified according to the recent AJCC (8th edition, 2017) staging system^27^. A summary of the patient clinical information, and histopathological data is presented in Table 1. Table 2 shows the clinic sites from which the tumors were obtained, the number of tumor samples analyzed, and the corresponding melanoma disease stages of donor patients. The cohort had a preponderance of male individuals (62%) and 63% of all patients were older than 60 years. Among patients with available overall survival (OS) information, nearly 47% survived less than 5 years. Several factors could have influenced this outcome, for instance, 56 patients had thicker primary tumors (Breslow > 1 mm), which are associated with short survival.^6^ In addition, 48 of these patients were diagnosed before BRAF/MEK targeted treatment and immune checkpoint inhibitor therapies were introduced, which have improved the management of MM disease and patient survival.^33^, ^34^, ^35^ Furthermore, 66% of the patients had stage I to III disease at the time of tumor specimen sampling, and 32% stage IV. Primary tumors were histopathologically classified as Nodular melanoma (NM; 30%), Superficial Spreading Melanoma (SSM; 25%), Acrolentiginous Melanoma (ALM; 5%), Lentigo Malignant Melanoma (LMM; 0.4%), Ocular (0.4%), and Mucosal (1%). Besides, BRAF mutational status was studied, and 49% of samples were found to be mutated. The V600E mutation accounted for 84% of the BRAF mutated cases, and the rest comprised mostly other amino acid substitutions at the V600 position (Table 1) Overall, the 505 analyzed tumor samples included 13% primary tumors, 64% regional lymph node metastases, and 22% of other metastases.

**TABLE 1:** Clinical features of patients and tumors included in the MM500 cohort

	Gender		Age at diagnosis	
	85 patients	Female	range	23‐96 years
Patients	143 patients	Male	median	64 years
(232)	4 patients	NA	81 patients	<60 years
			145 patients	>60 years
			6 patients	NA

	Disease stage		Overall survival	
	5 patients	I	range	56‐77,00 days
	13 patients	II	median ± STD	1228 ± 1344 days
	134 patients	III	65 patients	<5 years
	74 patients	IV	75 patients	≥5 years
	6 patients	NA	22 patients	unknown cause of death
			70 patients	NA

	Types		BRAF mutation status^‡)^	
	Primary tumors	64	wt	112
Tumor	Regional lymph node metastases	321	V600E	122
samples	Other metastases^†)^	112	V600K	16
(505)	NA	8	V600R	1
			V600A	1
			K601E	5
			NA	37

	Clinical classification		Breslow(mm)	
Primary	SSM	6	<1	1
tumors	NM	4	1.01‐2.00	1
(21)	ALM	3	2.01‐4.00	5
	NA	8	>4.00	14

†) These included cutaneous, subcutaneousand visceral metastases.

‡) BRAF mutation status of all tumorsanalyzed.

NA: Data notavailable.

**TABLE 2:** Clinic sites from where the samples were obtained

	BioMEL biobank, Lund University Hospital	Semmelweis University Hospital	Szeged University Hospital	Total
Patients	147	75	10	232
Tumors/Tumor samples	147/289	137/165	10/51	294/505
# primary tumors/# primary tumor samples	0	16/44	5/20	21/64
# metastatic melanomas/# metastatic melanoma samples	147/289	121/124	5/28	273/441
Tumor samples Stage I‐III	220	70	32	322
Tumor samples Stage IV	65	93	19	177
Tumor samples Stage NA	4	0	2	6

NA: Data not available.

### 2.2 Heterogeneity of melanoma tissues in MM500

**FIGURE 2:**
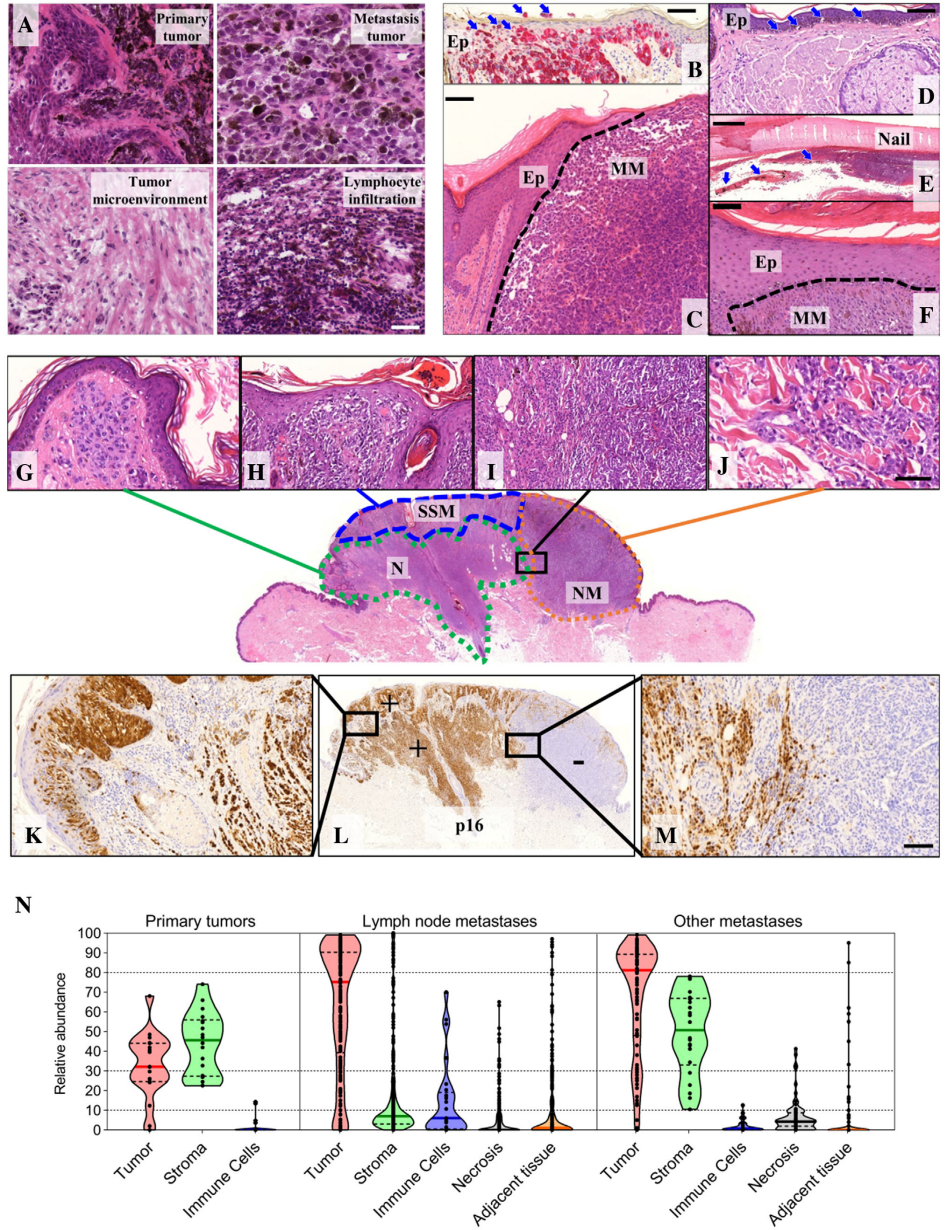
Both melanoma tissue and tumor‐derived stroma were collected for the study (A). Representation of the classical variants of melanoma subtypes as (B) superficial spreading melanoma with radial growth pagetoid tumor cells within the epidermis, transepidermal tumor cell elimination is also noted (arrows; Melan A stain highlights pagetoid cell by red discoloration), (C) nodular melanoma with solid extension and sharp border (dashed line) from the adjacent epidermis, (D) lentigo maligna with longitudinal atypical melanocytes along the dermoepidermal junction (arrows), and (E‐F) acral melanoma in subungual location. Ep: epidermis, MM: melanoma, bar indicates 100 μm and 500 μm in inserts of B‐C‐D‐F and E, respectively. Downstream inserts (G‐J) show different counterparts of a sequentially transformed melanocytic lesion from a nevus background (G) into superficial growth phase (H), then high grade vertical/nodular growth pattern (I‐J) with massive infiltrative activity downstream. The different regions can be highlighted by different expression of p16 protein as checkerboard pattern of the residual nevus (K‐left bottom) has changed to diffuse strong reactive p16 positivity (M‐left upper) in the transformed superficial spreading melanoma, however, there is already a complete loss of p16 in the high‐grade vertical growth melanoma part (L‐right half). (N) Violin plot representation with a summary of histological parameters evaluated for primary tumors, regional lymph node, and other metastases. N: nevus, SSM: superficial spreading transformed melanoma, NM: the nodular/vertical growth counterpart of the same tumor; bottom: IHC, DAB brown discoloration indicates p16 positivity; upper bar: 50 μm, bottom bar: 100 μm

Both melanoma tissue and tumor‐derived stroma were collected for the study. Representation of the classical variants of melanoma subtypes is shown in Figure 2A‐J. Primary tumors and metastatic tissue samples were characterized by histopathology including: percentage of (1) tumor cell, (2) stroma, (3) immune cells within the tissue, (4) necrosis and (5) adjacent tissue (Figure 2N). Melanoma cell content varied significantly (ANOVA test, FDR < 0.05) between primary tumors and both lymph nodes and other metastases. Immune cells presence in the samples was statistically higher (ANOVA test, *p*‐value < 0.05) in regional lymph node metastases compared to other tumor types, which were below 20%. Stromal components showed a significantly lower content (ANOVA test, *p*‐value < 0.05) in lymph node metastases than in primary tumors and metastases from other locations. In contrast, there were no significant differences between lymph node and other metastases in the contents of necrosis (*t*‐test *p*‐value = 0.193) and adjacent tissue (*t*‐test *p*‐value = 0.119).

The intratumor cell heterogeneity observed within the specimens is exemplified in the image from one of the melanoma patients (Figure 2G‐M).

**FIGURE 3:**
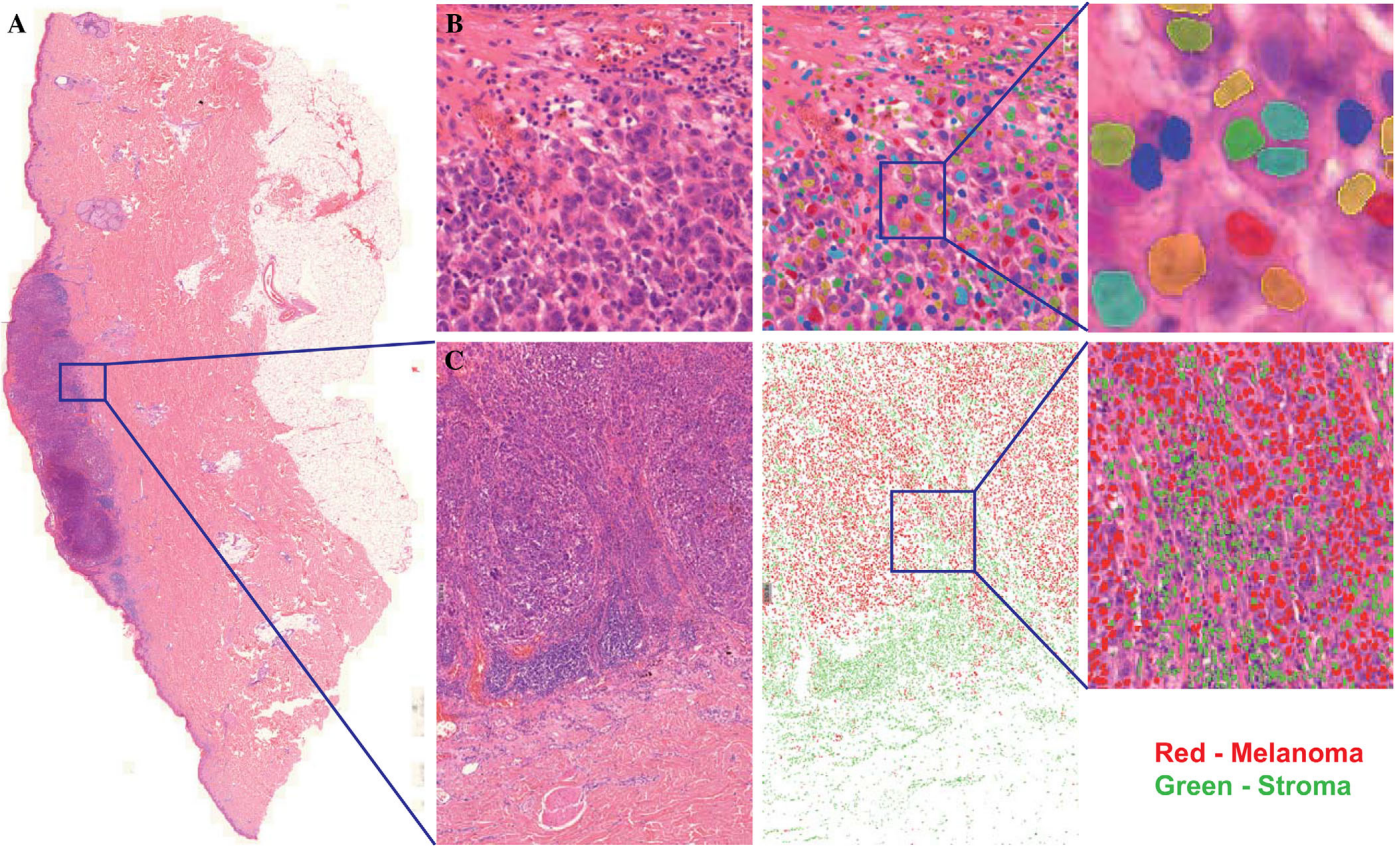
(A) Digitized melanoma tissue sample, (B) segmentation of every single cell in the tissue using a deep neural network method, (C) phenotypic classification of the tissue sample into malignant image captures from melanoma tumors in the pulmonary tract; (upper panel) microscope light image capture, (middle panel) image capture from H&E‐stained tumor tissue, and (lower panel) image capture of immunohistochemical staining Intelligent decision‐making system

DP is slowly gaining acceptance in routine diagnostics and can now be utilized both for frozen sections and paraffin tissue blocks.^24^, ^36^, ^37^ The utility of deep neural networks is approaching a level of accuracy similar to or even higher than analysis made by dermatologists and dermatopathologists.^38^, ^39^, ^40^ To further characterize the study tumor samples, a method for visualizing the heterogeneous melanoma morphology by DP was created, utilizing recent developments in machine learning. The combination of deep learning‐based single‐cell segmentation and phenotyping now for the first time enables digital dissection of melanoma samples at the resolution of a single cell (Figure 3A and B) and phenotype assignment to individual cells (Figure 3C).

**FIGURE 4:**
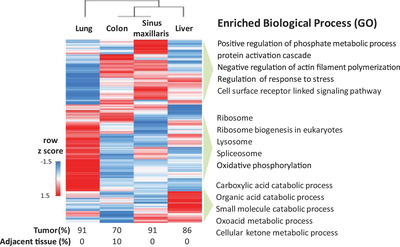
Heterogeneous protein profiles of multiple metastases removed from the same patient. Heatmap of unsupervised hierarchical clustering of 6699 protein abundance profiles for four surgically isolated melanoma metastases from distinct sites of the body in the same patient within the MM500 cohort. Gene ontology (GO) enrichment analysis based on protein expression showed marked differences in biological processes among the metastases. The figure displayed the five most significantly enriched different biological processes in each distinct site of metastasis. Tumor and adjacent tissue contents of the metastases are displayed at the bottom of the heatmap.

Investigation of the intratumoral heterogeneity of primary melanomas revealed that the clonal evolutionary pathways of the manifested metastases are very diverse; not just at the histological, but also at the molecular level.^41^, ^42^, ^43^ This is exemplified by four metastases from one patient, all displaying high tumor contents (≥70%) and low percentages of adjacent tissue (0‐10%). Here protein profiles showed marked differences across the metastases, with expressed proteins enriched in distinct biological processes (Figure 4). Interestingly, despite of the histological evaluation, the liver metastasis showed enrichment in processes that can be related to the tumor location.

### 2.3 Protein identification and melanoma proteome dynamics

**FIGURE 5:**
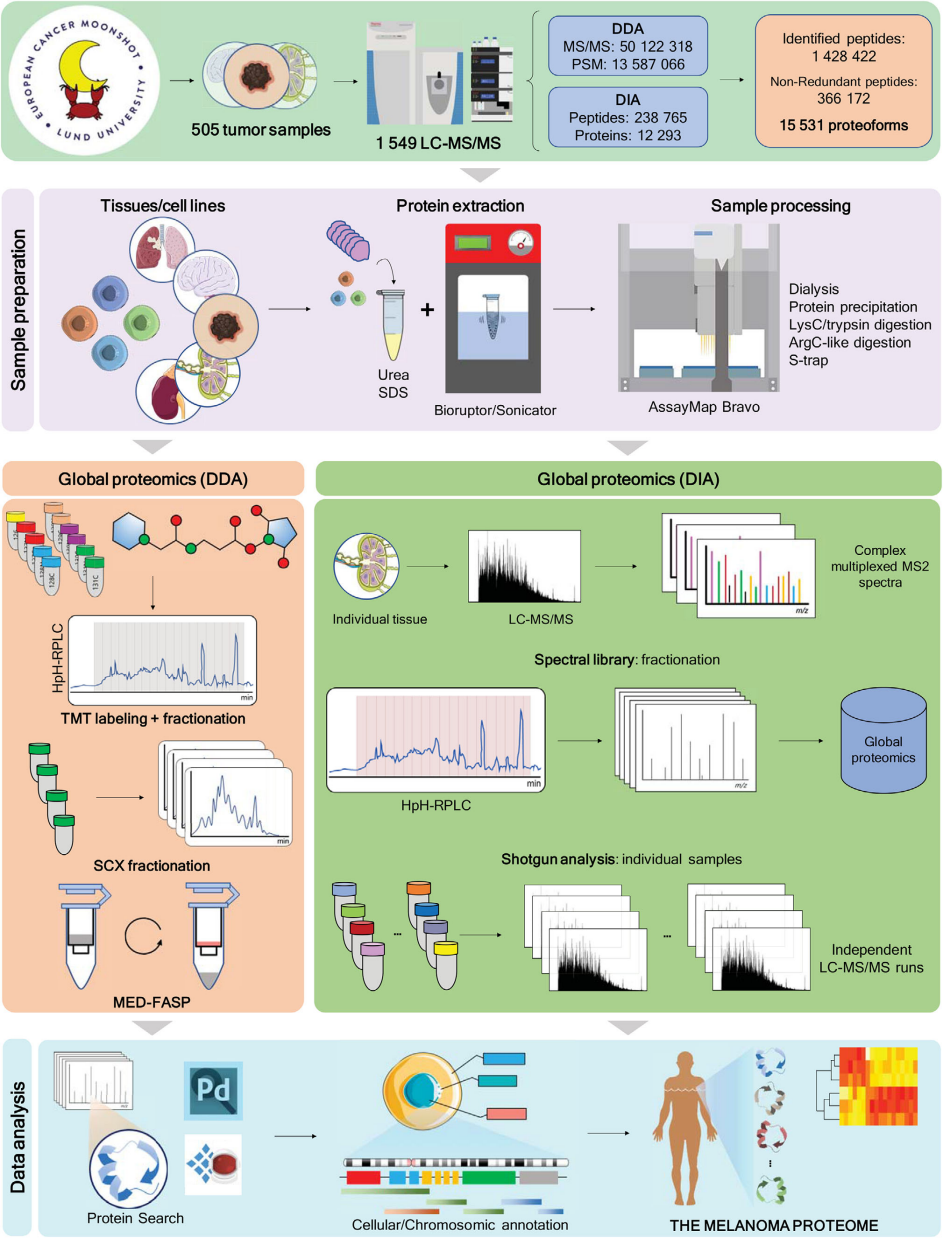
General scheme comprising the proteomic workflows used throughout the MM500 study. Upper panel) Overall results from the melanoma proteome as studied by the European Cancer Moonshot Lund Center, were more than 500 melanoma tissue samples and four cultured cell lines were analyzed. Sample preparation) Proteins were extracted in the presence of urea or SDS with the aid of a Sonifier or a Bioruptor. Manual or automatic enzymatic digestion protocols were carried on the protein mixtures depending on the agent used for protein extraction. Global proteomicsDDA‐MS) DDA data were generated from labeled or labeled‐free peptides. We analyzed TMT 11‐plex labeled peptides after high pH RP‐HPLC fractionation. Labeled‐free peptides were directly analyzed (shotgun proteomics), fractionated by SCX stepwise separation, or by the MED‐FASP method. Global proteomics DIA‐MS) MS/MS spectral libraries for DIA‐MS global proteomics were built out of DDA‐LC‐MS/MS data. This included shotgun analysis of the very same samples submitted to DIA‐MS, of other samples from melanoma tissues and cultured cells used in this metastudy, as well as the analysis of a mixture of these samples previously fractionated by high pH RP‐HPLC. Shotgun (analysis) Individual samples were submitted to LC‐MS/MS analysis either in DDA or DIA modes. Data analysis) The programs Proteome Discoverer and Spectronaut were used for protein identification and quantitation.

All tumor samples were submitted to the slicing procedure where up to 30 slices were selected for proteomics, while the first and last slices were saved for histological confirmation of melanoma tumor tissue. The strategies for sample processing, protein digestion, and MS data acquisition were summarized in Figure 5, where several state‐of‐the‐art procedures were optimized and/or implemented in our laboratory.^44^, ^45^, ^46^, ^47^, ^48^

**FIGURE 6:**
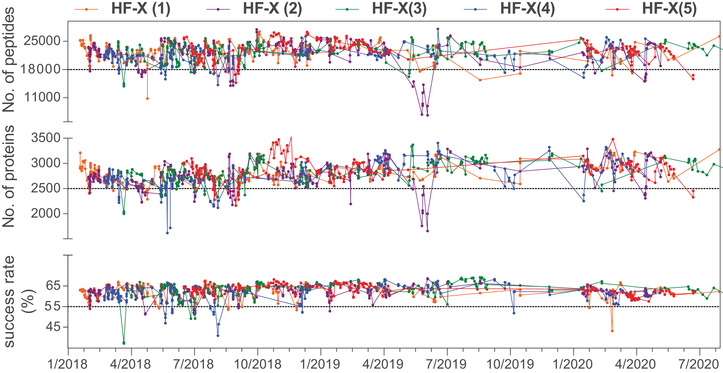
Quality metrics used for the systems suitability test during the MM500 metastudy. A total of 200 ng of Hela total protein digest were analyzed under the same chromatographic and MS parameters. The graph plotted the success rate (calculated based on the ratio between the PSMs and the total MS/MS), the number of peptides and proteins identified for each analysis across 2.5 years. Dash lines indicate the acceptable limits to continue analyzing the biologically relevant samples.

For protein quantification mainly two approaches were adopted, the label‐free and the isobaric labeling using tandem mass tag TMT‐11plex. The samples analyzed by the TMT approach were fractionated after labeling. Furthermore, for MS data acquisition two strategies were applied, the classical data dependent acquisition (DDA) approach and a data independent acquisition (DIA) strategy consisting of variable wide windows selected for MS/MS together with a spectral library for peptides and proteins identification. Samples were analyzed under a system suitability test designed to ensure the best performance of the LC‐MS instruments. Here, a standard peptide mixture from HeLa cells total protein digestion was used as a quality control sample to monitor different metrics (Figure 6).

In total, 1549 liquid chromatography‐tandem mass spectrometry (LC‐MS/MS) experiments were conducted with both melanoma tumor, and cultured cell line samples. These generated more than 51 million MS/MS spectra and resulted in approximately 13.6 million peptide‐to‐spectrum‐matches (PSMs) that successfully annotated 15,531 proteoforms, which included canonical proteins and isoforms (Supporting Information Table S1). Altogether we identified 12,878 protein‐coding genes (Supporting Information Table S2), represented by 3,66,172 peptides.

**FIGURE 7:**
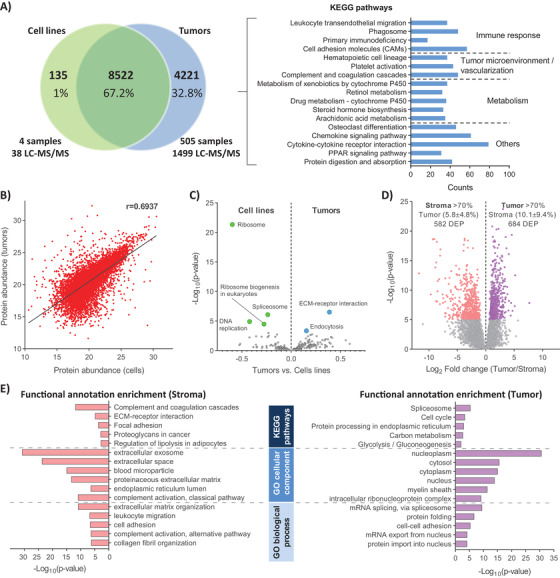
Comparison of different melanoma proteome profiles. (A) Venn diagram of protein‐coding‐genes identified in the metastudy in tumor samples and cultured cell lines, together with the number of analyzed samples, LC‐MS/MS experiments and KEGG pathway enrichment of proteins exclusively identified in the tumor samples. (B) Pairwise scatter plots of median abundance of commonly quantified proteins in melanoma tumor tissues and cultured cell lines. (C) Volcano plot of biological pathways significantly enriched when comparing the mean protein abundance between melanoma tumor tissues and cultured cell lines, according to 1D functional enrichment analysis provided by Perseus software.^70^ A false discovery rate (FDR) of 0.05 was set as the cut‐off for significance. (D) Volcano plot of DEP proteins between melanoma samples with high content of tumor or stroma cells. (E) Biological pathways significantly enriched for upregulated proteins in melanoma samples with high content of tumor or stroma cells

In cultured melanoma cell lines 10,511 proteoforms were identified and quantified, representing 8657 protein‐coding‐genes (Figure 7A, Supporting Information Table S3). Interestingly, 135 (1.5%) protein‐coding‐genes were exclusively identified in melanoma cell lines despite having significantly fewer analyzed samples, LC‐MS/MS experiments, and a lower number of identifications. We did not find any particular enrichment in pathways or Gene Ontology terms for this group of proteins. Out of the 135 proteins, 119 were found as transcripts in the TCGA database for melanoma, and 13 of the remaining proteins were detected in melanoma tumors either by immunohistochemistry or at mRNA level according to the Human Protein Atlas (HPA) (Supporting Information Table S5). Overall, there were only three proteins for which no evidence of expression was found in both databases and the analyzed tumor samples. Several factors might have influenced the result such as false‐positive identifications (1% in our study) or protein‐specific expression as an artifact due to cell culture conditions. In contrast, 4221 proteins were uniquely identified in the tumor samples, which can be attributed to the large diversity of cell types in the tissue, and the intra‐, and intertumor heterogeneity. (Figure 7A). The KEGG pathways enrichment analysis showed that most of these proteins are involved in immune system related pathways, or they are part of the extracellular space/vascular system (Figure 7A). These findings highlight the importance of studying the molecular profiles of melanoma in the context of the tumor tissue and its microenvironment, in order to better understand the mechanisms responsible for development and progression of the disease. Although established melanoma cell lines to some extent will mimic the disease, the changes imposed by interaction with the tumor microenvironment is very limited. However, the proteins commonly identified in both cell lines and tumors showed a significant correlation (Pearson *r* = 0.733, *p*‐value = 10e‐323) in their abundance profiles (Figure 7B), indicating that, melanoma cell lines also show tumor‐like relative protein levels. The differential proteome analysis between tumors and cell lines concludes that cell lines mainly upregulate pathways related to cell proliferation and in contrast, tumors mainly upregulate proteins involved in cell‐cell communication and endocytosis (Figure 7C).

The MM500 study included the analysis of melanoma samples with large variation in the cellular composition, which was evident from the histopathology characterization (Figure 2N). In particular, a subset corresponded to samples with low tumor cell content and more than 70% of stroma. The differences between this group and samples with the highest tumor cell content (>70%) were explored through multiple *t*‐test analysis (FDR 0.01). In total, 1266 proteins were regulated in relation to the cellular composition of the tumor tissues (Figure 7D). The functional annotation analysis showed that samples with high stroma content were, as expected, enriched in proteins involved in the extracellular matrix and its interaction with the cells, in addition to elements of the complement and coagulation cascade. Oppositely, tissue samples with high tumor cell content were significantly enriched in proliferation related pathways including metabolic pathway linked to rapid energy production (Figure 7E). These examples highlight the diversity in the disease presentation in melanoma, which can be captured by combining histopathology and proteomics.

The BRAF V600E mutation is present in 40‐60% of melanomas.^49^, ^50^ To investigate the differences in the proteome profiles on BRAF mutated and wild‐type (WT) melanoma tumors, we compared the protein expression of 48 samples with BRAF V600E mutation and 70 WT BRAF samples that were processed and measured under similar conditions. A statistically significant difference (*t*‐test, FDR 0.05) between the two groups was seen for only two proteins. A recent study, although with fewer samples, only found 17 differentially expressed proteins when compared the protein expression between BRAF mutated and WT tumors.^51^ The results suggest that tumors with the BRAF V600E mutation does not display a specific proteomic profile when compared to WT BRAF tumor. Nevertheless, we recently reported that the level of expression of the mutation does cause a different proteome profile within BRAF V600E positive tumors, which also correlates with differences in tissue morphology and patient outcome.^15^

### 2.4 Protein localization: cell and chromosome

**FIGURE 8:**
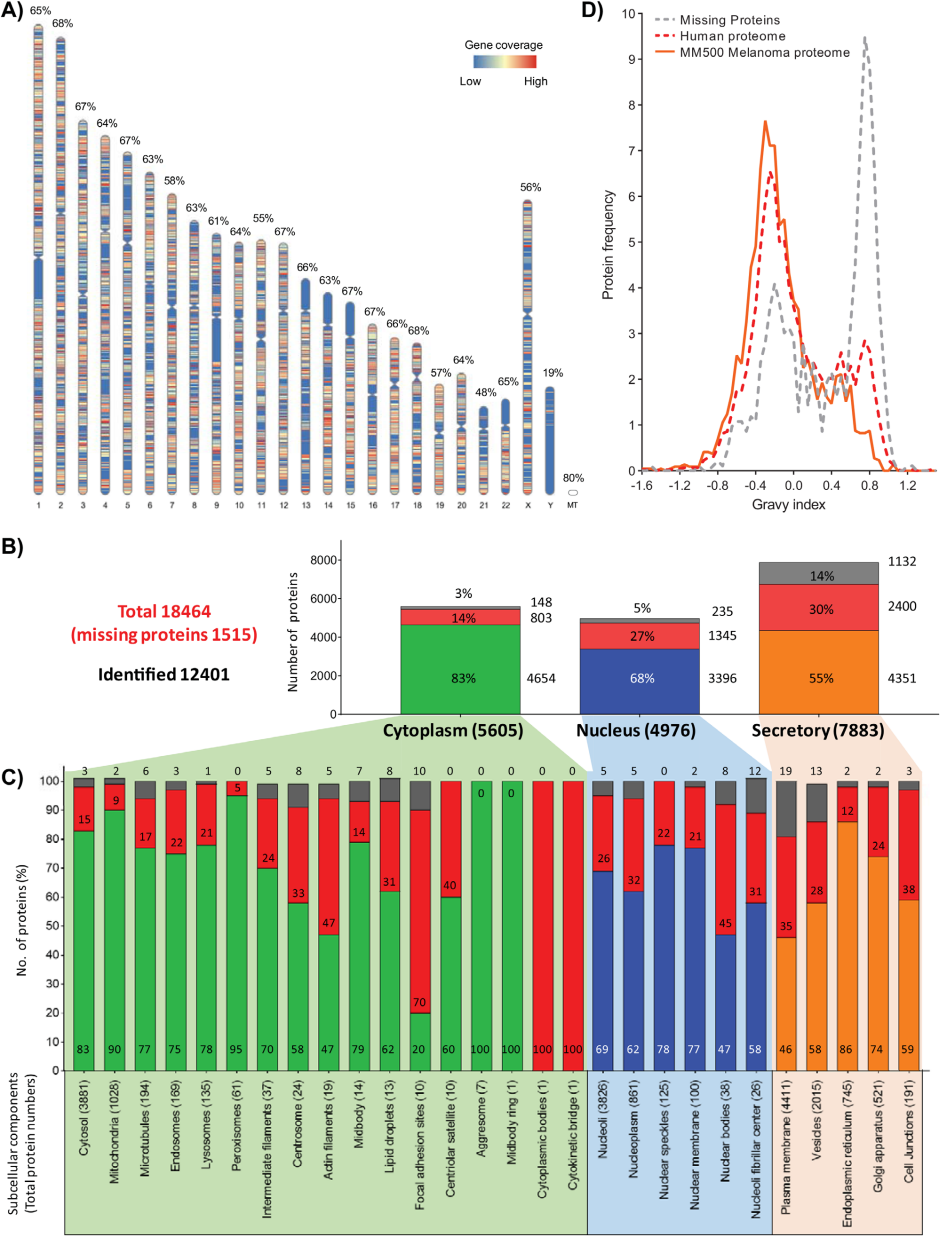
Protein localization: chromosome and cell. (A) Display of the melanoma proteome (13,219 protein‐coding‐genes) annotated to their respective chromosomal localization. Each colored heat‐map of the chromosomal ideogram indicates the gene density. The percentage of the total number of proteins expressed within each respective chromosome is also provided. (B) Melanoma proteome localization and its subcellular compartments, proteins allocated to the higher‐order groups; Cytoplasm/Nucleus/Secretory, and (C) subsequent detailed 27 localizations, respectively. The total number of proteins is in parenthesis nearby the corresponding name of the cellular and subcellular compartments. The bar graphs display the percentages in each localization represented by the Melanoma proteome (in green, blue, or orange color depending on the compartment), the fraction of the human proteome not covered (red), and the segment corresponding to the missing proteins (gray). (D) Gravy index enrichment plots of protein localized in the plasma membrane of the human proteome, the missing proteins, and the melanoma proteins identified in this metastudy

In the present study, we go one step deeper for the spatial localization of proteins, a critical step to understand biological functions.^52^, ^53^ An outline of the gene products of the 12,878 identified protein‐coding genes was constructed and organized to chromosome and subcellular localization (Figure 8).

Interestingly, most chromosomes (C) were evenly covered by the identified proteins (55to 68% in chromosomes, and 80% in mitochondrial genome) with the exceptions of chromosome 21 (48%) and the Y chromosome (19%) (Figure 8A). The chromosomal p arm has low, or no, gene density of expressed proteins for C13 to C15 and C21, and C22. A low density of expression was also observed in the q arm close to the centromere of C1, C9, and C16.

The identified proteins were distributed and categorized according to the Cell Atlas database from the HPA. From the interrogated melanoma data (Supporting Information Table S6), UniProt localized 11,141 proteins. Of the 1726 nonlocalized entries returned by the first search against UniProt, a further 1260 new protein localizations were determined from a complementary search against the HPA database with 240 enhanced, 60 supported, 575 approved, and 385 uncertain immunohistochemistry reliabilities. Based on the 19,773 predicted human protein‐coding genes approved by the Human Proteome Project of the Human Proteome Organization (HUPO)^54^ the total annotation for proteins identified in our study equates to 12,860 localizations. This includes 361 proteins with localization information confirmed by immunofluorescence images, and confocal microscopy and directly derived from SK‐MEL‐30 melanoma cultured cells. (Supporting Information Table S7).

The melanoma proteome was annotated into 26 different cellular localization comprising organelles, structures, and substructures. Figure 8B shows the main localization of proteins sorted by cellular compartments. The largest melanoma proteome localized to the cytoplasm with 4654 proteins, followed by 4351 secreted and 3396 nuclear proteins, which account for 83, 58, and 68%, respectively when considering the corresponding proteins of the human proteome annotated to these organelles.

The lower coverage of secreted proteins might be explained by the significant number of missing proteins in the human proteome that are classified as secreted. Currently, these proteins lack sufficient experimental data from mass spectrometry or other direct protein identification methods.^55^, ^56^

As detailed in Figure 8C, the proteins of 13 out of 17 cytoplasmic organelles and structures were covered, from 58 to 100%. The cytosol was the most populated with 3210 proteins (83%), and in the rest of the compartments, albeit with lower percentages, just a few dozens of proteins were not identified. A similar panorama was found for the nucleus where more than 68% of proteins from the six subcellular locations were identified. Within this organelle, nuclear body proteins were less represented with only 20 proteins that were not identified.

Interestingly, the plasma membrane accounts for the highest number of proteins (4411) among all the cellular compartments, and at the same time, it exhibited one of the lowest percentages of protein coverage in our melanoma protein data (49%, 2036 proteins). These proteins could be peripheral or integral membrane proteins, with the later known to possess hydrophobic transmembrane domains. Therefore, the grand average of hydropathicity (GRAVY) index was investigated for plasma membrane proteins from the melanoma proteome as well as for those classified as missing proteins (Figure 8D). For the different data sets, similar patterns were obtained, which indicated that the MM500 tissue analysis protocols covered almost the whole hydrophobicity spectrum of proteins. However, a decrease in frequency for the more hydrophobic proteins (GRAVY‐index ≥0.6) was seen in the melanoma dataset. Missing proteins in the secretory component were highly represented in the plasma membrane (31% of all missing proteins) and particularly towards the high end of the hydrophobicity range. Consequently, the missing proteins were a determinant factor in the lower coverage of plasma membrane proteins within the melanoma proteome. Altogether, the proteins identified here were classified and assigned to 26 of 28 distinct subcellular localization categories, representing 67% of the human proteome.

## 3 CONCLUSIONS AND FUTURE PERSPECTIVES

The histopathological characterization of more than 500 melanoma tumor samples showed a very heterogeneous disease, not only intertumor but also intratumor. A significant set of melanoma samples showed low levels of tumor cells, which in most cases correlated to high stromal content. Primary lesions presented a relatively lower tumor cell content compared to regional or distant metastases.

This MS melanoma proteome study identified 15,500 proteoforms, covering 65% of the total human proteome. Also, among the large number of cytoplasmic proteins identified more than 2000 membrane proteins were detected, including receptors and ligands usually present in low copy numbers. Combining the detailed molecular characterization of expression with histopathology contributes to the pathologist definition of tumor samples. The presented outline of the melanoma proteome can now form a basis for hypothesis building within the diagnosis, treatment, drug discovery, and drug development. For example, the annotations of specific key regulating proteins and their respective functions, cellular localizations, whether intracellular, or nuclear, can be associated with the mode of action of possible therapeutic agents. Information of the abundance and origin of expressed proteins; for example, in primary tumors, or metastasis within the lymph nodes, lung, brain and other organs, coupled to detailed clinical phenotyping, complement the investigation. Taken together, the reported melanoma proteome data will be vital for generating a better understanding of the disease and the mechanisms that drive the progression.

## 4 MATERIAL AND METHODS

### 4.1 Chemicals and reagents

Dithiothreitol (DTT), iodoacetamide, ammonium bicarbonate (Ambic), ammonium hydroxide, sodium docecylsulphate (SDS), Trifluoroacetic acid (TFA), sodium deoxycholate (SDC), tris(hydroxymethyl)aminomethane (Tris), formic acid, and urea were purchased from Sigma–Aldrich (St. Louis, MO, USA). Triethylamonium bicarbonate (TEAB) and hydroxylamine were from Thermo Fisher Scientific. Water and organic solvents were all LC–MS grade and supplied by Merck (Darmstadt, Germany) or (Thermo Fisher Scientific). Endoproteinase Lys‐C was obtained from Wako (Osaka, Japan) and sequencing‐grade modified trypsin was purchased from Promega (Madison, WI, USA). Cell lines SK MEL2 (HTB‐68), SK MEL28 (HTB‐72), and RPMI‐7951 (HTB‐66) were obtained from the American Type Culture Collection (ATCC).

### 4.2 Tissue specimens

A total of 505 patient tumor samples (Figure 1 and Table 1 & 2) obtained from University clinics in Sweden and Hungary, was included in the study. All studies were approved by the local ethical committees, including the Regional Ethical Committee at Lund University, Southern Sweden (DNR 191/2007, 101/2013 (BioMEL biobank), 2015/266 and 2015/618), Semmelweis University, Hungary (191‐4/2014), University of Szeged, Hungary (MEL‐PROTEO‐001). All patients provided written informed consent. The malignant melanoma, primary and metastatic tissue, samples were snap‐frozen within 30 min after surgical resection with a small amount of isopentane in liquid nitrogen or put on dry ice within 20 min of surgery. Multiple pieces were collected from most of the tumor specimens. The source of the analyzed tissue samples and patients who provided them was as follows: Lund University Hospital 289 samples from 147 patients, Semmelweis University Hospital 165 samples from 75 patients, and Szeged University Hospital 51 samples from 10 patients.

Fresh‐frozen tissues collected at Lund University Hospital were stored in the Melanoma biobank, BioMEL, Region Skåne, Sweden. Tissues collected at sites in Hungary were stored at the respective biobanks of Semmelweis University and the University of Szeged. Samples transportation from Hungary was carried out in liquid nitrogen. In the case of FFPE samples, the fixation of tumor tissues was performed right after surgery with 4% buffered formaldehyde. Samples were then dehydrated in xylene/alcohol solution and embedded into paraffin. FFPE samples were transferred and stored at room temperature. Sections of 10 μm were used for further analysis. The study has been performed in compliance with GDPR.

All tumors were processed with integrated Biobanking consolidations within all involved medical centers. The workflow was built according to Swedish biobanking laws and best practices and guidelines provided by the BBMRI‐ERIC, ESBB, and ISBER (https://www.bbmri‐eric.eu/services/quality‐management).^57^ The process flow enabled rapid sample handling whereby collected tissues were stored at an ultra‐low temperature in a biobank at a cycle time of approximately 20 min. Using the same data management system and database reconnaissance, sample integrity was ensured via electronic surveillance. The patient and sample processing workflow and protocols for the Semmelweis and Szeged cohorts were transferred and interfaced with the RedCap database, (https://www.project‐redcap.org/).^58^

### 4.3 Cell cultures

All melanoma cell lines (SK‐MEL‐2, SKMEL28, and VMM1) were purchased from ATCC and cultured and maintained at standard conditions and recommendations by the manufacturer. In detail, SK‐MEL‐2 and SKMEL28 were maintained with DMEM (Dulbecco's modified Eagle's medium) supplemented with 10% fetal bovine serum (FBS) and penicillin‐streptomycin (P/S). VMM1 were maintained with RPMI‐1640 supplemented with 10% FBS and P/S. All cells were maintained at 37°C in a humidified 5% CO_2_ incubator.

### 4.4 Histopathological analysis

Stepwise sectioning of the tissues was performed using a cryostat and setting the slices thickness at 10 μm. On average, three sections were evaluated. Tissue sections were placed on glass slides, stained with hematoxylin and eosin, and then placed in an automated slide scanner system (Zeiss Mirax, Jena, Germany). The slides were then evaluated for tissue content: tumor, necrosis, stroma, tissue, and adjacent background tissue—mostly lymphatic cells—lymph node area.^52^

#### 4.4.1 Statistical analysis

One‐way analysis of variance (ANOVA) followed by Tukey post‐hoc analysis for multiple comparisons and paired *t‐*test, were used to determine the statistical significance of histological parameters between the different tumor types.

### 4.5 Sample preparation for mass spectrometry

#### 4.5.1 Deparaffinization of FFPE tissue

The FFPE tissue sections were incubated with 1 mL of 1:50 diluted EnVision™ FLEX Target Retrieval Solution High pH (Agilent Dako) at 97˚C for 10 min (500 RPM). Incubation was followed by a brief centrifugation at 14,000 g at 4˚C for 3 min, removal of the EnVision solution and the paraffin. These steps were repeated until complete paraffin removal as previously described.^59^

#### 4.5.2 Protein extraction

For fresh‐frozen tissues, the lysis buffers contained 100 mM ammonium bicarbonate or 100 mM Tris pH 8.6 and up to 6 M Urea or 2% SDS. Lysates were generated by sonication in an ice batch using a Branson Sonifier 250 (output 4, 10% duty cycle) or using the Bioruptor plus, model UCD‐300 (Dieagenode) for 40 cycles. Each cycle consisted of 15 s at high power and 15 s without sonication at 4°C. Samples were centrifuged at 10,000 g and 4°C for 10 min and the supernatants were transferred into a new tube and the pellet was discarded.

In the case of FFPE tissue samples, the protein extraction was performed by adding 100 mM TEAB containing 25 mM DTT and 10w/v% SDS pH 8. The samples were incubated at 99˚C for 1 h with shaking (500 RPM) and sonicated in the Bioruptor® Plus UCD‐300 (Diagenode) for 40 cycles (15 s on and 15 s off) at 4˚C, followed by centrifugation at 20,000 g at 18˚C for 10 min.

#### 4.5.3 Protein determination

The protein in each one of the samples was determined using a colorimetric micro BCA Protein Assay kit (Thermo Fisher Scientific, Rockford, IL) following the manufacturer's instructions.

#### 4.5.4 Protein digestion

Proteins were reduced with 10 mM DTT for 1 h at 37°C and alkylated with 40 or 50 mM iodoacetamide for 30 min, in the dark, at room temperature. Proteins were digested overnight with trypsin or Lys‐C and trypsin using published and optimized protocols including buffer exchange^47^, ^60^ or urea in‐solution digestion^46^ which comprised automated sample handling.^44^ SDS was removed from the samples by the MED‐FASP method^48^ or by ethanol precipitation.^61^ The later was followed by protein solubilization in 50 mM Ambic with 0.5 SDC (Sodium deoxycholate) and trypsin digestion. For acetylation analysis, the samples were processed and digested as previously described.^61^ (See Material and Methods of Supporting Information)

FFPE derived protein extracts were digested using the S‐trap method following the manufactures’ instructions with a few modifications as reported.^59^

#### 4.5.5 TMT 11 plex labeling

TMT11 plex labeling was performed according to manufacturer's instructions.

#### 4.5.6 Peptide fractionation

TMT‐11 and labeled‐free peptides were separated by basic pH reversed‐phase liquid chromatography (HpH RP‐HPLC) on a Phenomenex Aeris C8 column (100 ×  2.1 mm, 3.6‐μm particles) using an Agilent 1100 HPLC system and a gradient with solvent A 20 mM ammonium formate (pH 10) and solvent B 80% can—20% water containing 20 mM ammonium formate (pH 10). Labeled‐free peptides were also fractionated by strong cation exchange (SCX) using Microspin columns (MA SEM HIL‐SCX, 10‐100 μg capacity, The Nest group Inc., South Borough) in stepwise‐elution.^47^, ^62^

#### 4.5.7 Peptide desalting

Enzymatic digestions were quenched by adding formic acid to a final concentration of 1%. Proteolytic peptides were desalted prior to LC‐MS/MS experiments. We used C18‐microcolumns (The Nest Group, MA, USA) following the manufacturer's instruction, or the AssayMAP Bravo platform using the peptide cleanup v2.0 protocol with C18 cartridges (Agilent, 5 μL bed volume). Peptides were eluted in 80% ACN, 0.1% TFA, dried on a Speedvac and dissolved in 0.1% formic acid or 0.1% TFA. Peptides generated by digestion with SDC protocol or on the S‐traps were directly analyzed by LC‐MS/MS without desalting.

#### 4.5.8 Peptide determination

The peptide quantity in each sample and fraction was determined using the Pierce Quantitative Colorimetric Peptide Assay according to the instructions provided by the manufacturer.

### 4.6 LC‐MS/MS analysis

We used two main LC‐MS/MS setups. System 1 comprised an Easy nLC‐1000 (Thermo Fisher Scientific) coupled to a Q Exactive Plus mass spectrometer (Thermo Fisher Scientific). Here the peptides ( ´∼1 μg) were initially loaded onto a trap column (Acclaim PepMap 100 precolumn, 75 μm i.d. × 2 cm, C18, 3 μm, 100 Å; ThermoFisher Scientific) and then separated on an analytical column (EASY‐Spray column, 75 μm i.d. × 25 cm, PepMap RSLC C18, 2 μm, 100 Å; ThermoFisher Scientific). System 2 comprised an Ultimate 3000 nLC (Thermo Scientific Bremen Germany) coupled to a Q Exactive HF‐X mass spectrometer (Thermo Scientific). For this case the peptides (´∼1 μg) were loaded in a trap column (Acclaim1 PepMap 100 pre‐column, 75 μm, 2 cm, C18, 3 m, 100 Å, Thermo Scientific, San José, CA) and then separated on an analytical column (EASY‐Spray column 25 or 50 cm, 75 μm i.d., PepMap RSLC C18, 2 μm, 100Å, Thermo Scientific). Both systems used a flow rate of 300 nL/min and a water/ACN gradient in 0.1% formic acid and samples were measured in DDA and DIA modes. The DIA‐MS Spectral library was built out of DDA‐LC‐MS/MS analyses of samples from tissue and cultured cell origin, with spiked in iRT peptides (Biognosis AG). This also included the analysis of a mixture of samples previously fractionated by HpH RP‐HPLC.

### 4.7 Data analysis

#### 4.7.1 Peptide and protein identification and quantitation in DDA‐MS experiments

Raw DDA‐LC‐MS/MS files were analyzed with the Proteome Discoverer™ Software (Thermo Scientific™) against Uniprot Human dataset to which were added Fasta format protein sequences of known driver mutations of Melanoma disease.^63^ The search engine Sequest HT was used for peptide identification. Carbamidomethylation was set as a static modification as well as TMT 6plex (+229.1629 Da) at peptide and lysine *N*‐termini as well as lysine ε‐amino for labeling experiments. Oxidation of methionine residues and acetylation at protein *N*‐termini were selected as dynamic modifications. Precursor and fragment mass tolerance was set as 20 ppm and 0.02 Da, respectively, and two missed cleavages were allowed for peptides. The Minora node was included in the search for identification using retention time alignment and the match‐between‐runs features. For label‐free experiments, the quantification was carried out using the TOP3 method where the protein abundance is reported as the mean of the three highest peptides (unique and razor) areas measured for each protein. For TMT labelling experiments, protein abundances were calculated as the summed areas of reporter ions considering unique peptides. Identification and sorting of unique peptides were carried using the neXtProt tool “Peptide uniqueness checker” (https://www.nextprot.org/tools/peptide‐uniqueness‐checker).^64^

#### 4.7.2 Peptide and protein identification and quantitation in DIA‐MS experiments

A Global proteomics spectral library was generated from DDA experiments as described above. Raw files were converted to HTRMS files with a special converter provided by Biognosys AG and searched in the Spectronaut X platform (Biognosis AG) against the *Homo sapiens* database from Uniprot containing isoforms. Dynamic retention time prediction was selected to enable nonlinear alignment of precursor retention times between the (iRT, normalized retention time) spectral library and the DIA‐MS data by segmented regression. The following parameters were used: cysteine carbamidomethylation (+57.0215 Da) as fixed modification and methionine oxidation (+15.9949 Da), *N*‐terminal acetylation (+42.0105 Da) as dynamic modifications. A maximum of two missed cleavages were accepted. Precursor mass tolerance was set to 10 ppm and for the MS/MS fragments it was set to 0.02 Da. Filtering was performed at a 1% false discovery rate (FDR) for all the peptides and proteins that were used to construct the spectral library. The resulted library containing identified spectra for 220,360 peptides representing 12,293 proteins. The software computed MS1 peptide abundance as the summed precursor XIC (Extracted‐Ion Chromatogram, from the monoisotopic precursor ion plus isotopic envelope). The protein abundance resulted from the average of the top three most intense precursor ions corresponding to unique and razor peptides.

### 4.8 Localization of melanoma proteins and chromosomal distribution

The complete melanoma dataset of 12,878 proteins (Supporting Information Table S1) was confronted on 2020/09/18 with the UniProt database (https://www.uniprot.org, UniProt release 2020_03)^65^ for the initial spatial localization of proteins. Proteins not localized in the UniProt database were taken to a second search on the same date in the Human Proteome Atlas databank version 19.3 (https://www.proteinatlas.org/humanproteome/cell/organelle)^66^ for complementary subcellular location. All UniProt levels of evidence, for example, protein, transcript, inferred from homology and predicted, as well as the immunohistochemistry reliabilities of HPA, namely, Enhanced, Supported, Approved, and Uncertain were accepted for search, considering that identification by MS confirmed the presence of the gene product. Both databanks were searched for main subcellular localization only. Structures and organelles were grouped according to the HPA Human Proteome nomenclature and Thul et al.^52^ Based on genomic gene annotation and its chromosomal position, the R package RIdeogram^67^, ^68^ was used to map gene density heatmap on the chromosomes and its coverage percentages, respectively.

### 4.9 Protein normalization and analysis

The results from protein identification and quantification were imported into Perseus software version 1.6.14.0.^69^ Data were normalized by log2 transforming the protein intensities, and standardization was performed by subtracting individual values by the median in each sample. The proteins showing less variability across all batches that were identified in 100% of the samples were used to correct the abundance differences between batches. To do that, individual protein intensities in each batch were subtracted by the median abundance of selected proteins in the specific batch. After correction, the median abundance for each protein across all samples was calculated and reported as the relative abundance in our melanoma proteome. The statistical analyses were also performed using the Perseus software.

## Supporting information

Supporting information

Tables

## Data Availability

The data that support the findings of this study are openly available in ProteomeXchange at http://www.proteomexchange.org/, reference numbers PXD001725, PXD001724, PXD009630, PXD017968, and PXD026086 and will be complemented by the addition of data from the study. Tables 1 and 4 of Supporting Information are available at https://github.com/rhong3/TCGA_melanoma/tree/master/Supporting%20Information%20tables.
